# Oral Formulation of Angiotensin-(1-7) Promotes Therapeutic Actions in a Model of Eosinophilic and Neutrophilic Asthma

**DOI:** 10.3389/fphar.2021.557962

**Published:** 2021-03-08

**Authors:** Giselle Santos Magalhães, Juliana Fabiana Gregório, Arthur Tonani Pereira Cançado Ribeiro, Isis Felippe Baroni, Ana Victoria de Oliveira Vasconcellos, Gabriela Pansanato Nakashima, Isabel Fusaro Aguiar Oliveira, Natália Alves de Matos, Thalles de Freitas Castro, Frank Silva Bezerra, Ruben D. Sinisterra, Vanessa Pinho, Mauro Martins Teixeira, Robson Augusto Souza Santos, Maria Glória Rodrigues-Machado, Maria José Campagnole-Santos

**Affiliations:** ^1^ Department of Physiology and Biophysics, National Institute of Science and Technology in Nanobiopharmaceutics, Biological Sciences Institute, Federal University of Minas Gerais, Belo Horizonte, Brazil; ^2^ Post-Graduation Program in Health Sciences, Medical Sciences Faculty of Minas Gerais, Belo Horizonte, Brazil; ^3^ Laboratory of Experimental Pathophysiology, Department of Biological Sciences, Institute of Exact and Biological Sciences, Federal University of Ouro Preto, Ouro Preto, Brazil; ^4^ Chemistry Department, Institute of Exact Sciences, Belo Horizonte, Brazil; ^5^ Department of Morphology, Biological Sciences Institute, Federal University of Minas Gerais, Belo Horizonte, Brazil; ^6^ Department of Biochemistry and Immunology, Biological Sciences Institute, Federal University of Minas Gerais, Belo Horizonte, Brazil

**Keywords:** resolution of inflammation, eosinophilic inflammation, neutrophilic inflammation, allergic lung inflammation, LPS, renin–angiotensin system 4

## Abstract

The presence of eosinophils and neutrophils in the lungs of asthmatic patients is associated with the severity of the disease and resistance to corticosteroids. Thus, defective resolution of eosinophilic and neutrophilic inflammation is importantly related to exacerbation of asthma. In this study, we investigated a therapeutic action of angiotensin-(1-7) (Ang-(1-7)) in a model of asthma induced by ovalbumin (OVA) and lipopolysaccharide (LPS). Balb-c mice were sensitized and challenged with OVA. Twenty-three hours after the last OVA challenge, experimental groups received LPS, and 1 h and 7 h later, mice were treated with oral formulation of Ang-(1-7). On the next day, 45 h after the last challenge with OVA, mice were subjected to a test of motor and exploratory behavior; 3 h later, lung function was evaluated, and bronchoalveolar lavage fluid (BALF) and lungs were collected. Motor and exploratory activities were lower in OVA + LPS-challenged mice. Treatment with Ang-(1-7) improved these behaviors, normalized lung function, and reduced eosinophil, neutrophil, myeloperoxidase (MPO), eosinophilic peroxidase (EPO), and ERK1/2 phosphorylation (p-ERK1/2) in the lungs. In addition, Ang-(1-7) decreased the deposition of mucus and extracellular matrix in the airways. These results extended those of previous studies by demonstrating that oral administration of Ang-(1-7) at the peak of pulmonary inflammation can be valuable for the treatment of neutrophil- and eosinophil-mediated asthma. Therefore, these findings potentially provide a new drug to reverse the natural history of the disease, unlike the current standards of care that manage the disease symptoms at best.

## Introduction

Respiratory infections, most frequently those caused by Gram-negative bacteria, especially *Haemophilus influenzae*, *Mycoplasma pneumoniae*, and *Chlamydia pneumoniae*, are associated with asthma induction, progression, and exacerbation (Kumari et al., 2015; Hadjigol et al., 2020). Experimental use of lipopolysaccharide (LPS), one of the main components of the outer membrane of Gram-negative bacteria, has provided information on the effects of the inflammatory response to bacterial infection (Kumari et al., 2015; Hadjigol et al., 2020). Pulmonary inflammation induced by LPS promotes intense recruitment of neutrophils, increases airflow obstruction and edema, and exacerbates mucus production (Hauk et al., 2008; Hadjigol et al., 2020). Increased neutrophil levels have also been found in asthmatic patients who present poor response to inhaled corticosteroids (Green et al., 2002; Hadjigol et al., 2020). Corticosteroid-resistant asthma presented an increase in LPS in the bronchoalveolar lavage fluid (BALF) and inflammatory patterns characteristic of the activation of this endotoxin (Goleva et al., 2008). In this regard, Lowe et al. (2015) showed that guinea pigs with eosinophilic pulmonary inflammation induced by ovalbumin (OVA) presented a prolongation of pulmonary inflammation and reduction in corticosteroids sensitivity, after exposure to LPS. Hadjigol et al. (2020) also reported that exacerbations in an LPS-induced asthma model are associated with steroid resistance.

Studies showed that the combination of OVA and low doses of LPS promotes marked infiltration of eosinophils and neutrophils in the lungs, with increased Th2 cytokine production and more severe bronchoconstriction (Lowe et al., 2015; Camargo et al., 2017; Hadjigol et al., 2020). A recent study suggested that the presence of both eosinophils and neutrophils in the lungs contributes to exacerbation of asthma, as well as the interaction of the inflammatory response mediated by these cells (Camargo et al., 2017). Accumulation of eosinophils in the lungs is one of the main characteristics of allergic asthma (Felton et al., 2014; Nakagome and Nagata, 2018). Therefore, the inflammatory mediators released by these cells and the increase in their survival can induce damage to the lung tissue, leading to remodeling and impairment of organ function (Felton et al., 2014; Jasper et al., 2019). These changes represent failure to resolve the inflammatory process and induce chronicity of the disease (Felton et al., 2014; Jasper et al., 2019). Neutrophilic airway inflammation has been associated to severe, chronic, and acute forms of asthma, such as steroid-insensitive asthma, acute exacerbation of asthma, and occupational asthma (Green et al., 2002; Lowe et al., 2015; Gao et al., 2017; Hadjigol et al., 2020). Activated neutrophils might release cytokines, metalloproteinase, elastase, and myeloperoxidase (MPO) responsible for pulmonary damage (Jasper et al., 2019). Thus, in view of the worsening of symptoms induced by the infiltration of eosinophils and neutrophils in the lungs, as well as their relation to the development of insensitivity to corticosteroids, new therapeutic approaches are still in need to be investigated for asthma treatment.

Activation of the angiotensin-converting enzyme 2 (ACE2), angiotensin-(1-7) (Ang-(1-7)), and Mas receptor pathway (ACE2/Ang-(1-7)/Mas) of the renin–angiotensin system (RAS) induces anti-inflammatory, antiproliferative, antifibrotic, and proresolving effects in several acute and chronic inflammatory conditions (Galvão et al., 2019; Santos et al., 2018). Over the past few years, our research group has provided strong evidence that activation of the Mas receptor by Ang-(1-7) or by a nonpeptide agonist promoted beneficial effects in the lung (Rodrigues-Machado et al., 2013; Magalhães et al., 2015; Magalhães et al., 2016; Magalhaes et al., 2018; Bastos et al., 2020). In previous studies, we have shown effects in the prevention, treatment, and resolution of allergic lung inflammation (Rodrigues-Machado et al., 2013; Magalhães et al., 2015; Magalhães et al., 2016). Also, we observed that malfunctioning of the ACE2/Ang-(1-7)/Mas axis intensified inflammation and pulmonary remodeling (Magalhães et al., 2016). Here, we evaluated the potential of an oral formulation of Ang-(1-7) to reduce inflammation and lung damage in a model of eosinophilic and neutrophilic asthma.

## Methods

### Animals

All animal care and experimental procedures were approved by the Ethics Committee for Animal Experimentation (CEUA) of the Federal University of Minas Gerais (UFMG), Brazil (protocol #320/2018). Animals were from the animal facility of UFMG, Centro de Bioterismo (CEBIO), housed under a 12/12 h light-dark cycle (lights on at 06:00 h) with free access to standard chow and water. The mice (6–8 weeks of age, weighing 20–25 g) were randomly allocated into three experimental groups: 1) control group (CTRL; phosphate-buffered saline (PBS); *n* = 11); 2) sensitized and challenged with ovalbumin (OVA) and LPS (OVA + LPS; *n* = 13); and 3) sensitized and challenged with OVA and LPS and treated with Ang-(1-7) (OVA + LPS + Ang-(1-7); *n* = 13).

### Allergic Lung Inflammation Induced by OVA and LPS

**FIGURE 1:**
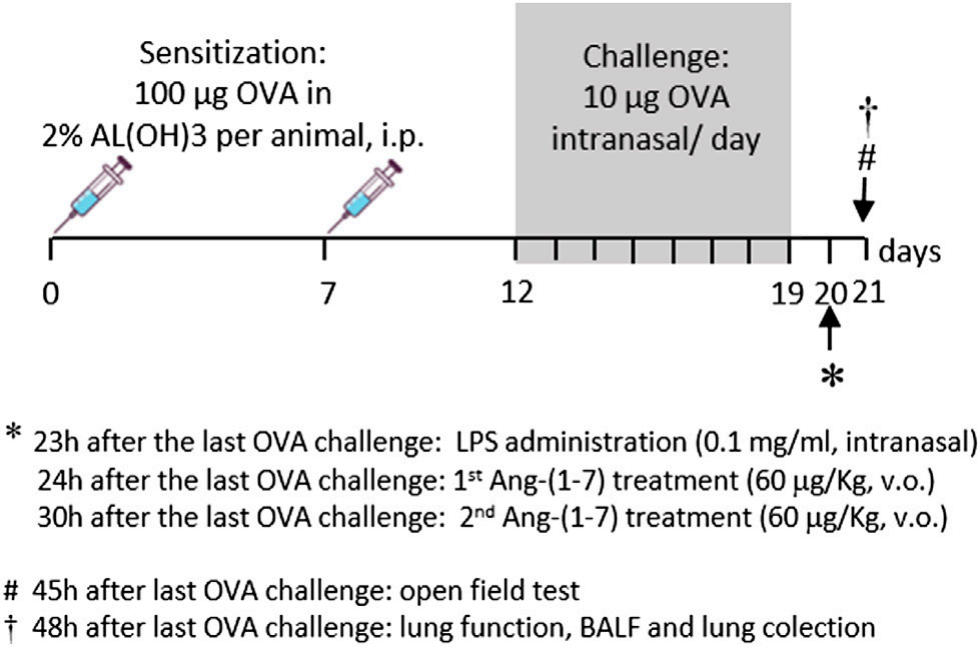
Time line of protocol used in the present study. Sensitization was made by two intraperitoneal injections of OVA (100 µg/kg in AlOH, 2%) at a 7-day interval. From the 12th day of protocol, sensitized mice received OVA challenge every day until the 19th day (10 μg, intranasal). Twenty-three hours after the last OVA challenge, experimental groups received LPS (0.1 mg/ml, intranasal). Mice were treated with Ang-(1-7) twice (1 h and 7 h after LPS). On the next day, 45 h after the last OVA challenge, mice were subjected to the test of motor and exploratory behaviors, and 3 h later, lung function was evaluated. Next, bronchoalveolar lavage fluid and lungs were collected.

The protocol used in this study is shown in Figure 1. Mice received two intraperitoneal injections of OVA (100 μg of OVA in 2% aluminum hydroxide diluted in PBS in a total volume of 0.2 ml via intraperitoneal injection, i.p.) at a 7-day interval (days 0 and 7). From the 12th day, mice of the sensitized groups were challenged daily with intranasal OVA (20 µl of solution containing 10 μg of OVA diluted in PBS) until the 19th day. The CTRL group received PBS on the days 0 and 7 (i.p.) and was challenged with PBS at the same timepoints (Magalhaes et al., 2018). Intranasal injections were made under ketamine (100 mg/kg) associated with xylazine (20 mg/kg; Rhobifarma Indústria Farmacêutica Ltd., Brazil) anesthesia. Allergic pulmonary inflammation was exacerbated by intranasal administration of LPS (20 µl of PBS + 0.1 mg/ml *Escherichia coli* 0127: B8, Sigma-Aldrich, St. Louis, MO, United States), 23 h after the last OVA challenge (20th day) under ketamine/xylazine (100 and 20 mg/kg, respectively) anesthesia (Camargo et al., 2017).

### Treatment With Ang-(1-7)/HP-β-CD

Animals of the OVA + LPS + Ang-(1-7) group were treated twice (6 h apart; 24 and 30 h after the last OVA challenge) with Ang-(1-7) (60 μg/kg included in 92 μg/kg of HP-β-CD orally). Both CTRL and OVA + LPS groups received 92 μg/kg of HP-β-CD (empty cyclodextrin) via gavage at these same timepoints (Magalhaes et al., 2018).

### Open Field Testing

Forty-five hours after the last challenge, an open field test was performed to evaluate the locomotor and exploratory activities of the animals, as described by Bastos et al. (2020). Briefly, the animals were adapted to the room where the test would be performed. Each mouse at a time was individually placed in a box (50 × 50 × 30 cm) of PVC material opaque white. The floor area of the box was divided into quadrants of 10 × 10 cm. The mouse was placed in a corner of the box and observed for 10 min. The total number of crossed quadrants (crossing) and the number of times the mouse rose on its hind legs (rearings) were counted by an independent researcher.

### Lung Function, BALF, and Tissue Collection

In five animals of each group, lung function was evaluated 48 h after the last challenge. For this, mice were individually anesthetized with a mix of ketamine (100 mg/kg) and xylazine (20 mg/kg). After that, a small neck incision was made, and the trachea was cannulated with an 18G catheter coupled to a 2 cm long tube to connect the trachea to the flow head (MLT1L). Breathing was monitored with the use of a differential pressure transducer (Spirometer, ADInstruments, Bella Vista, NSW, Australia). The signal was then amplified (Octal Bridge Amp model ML228; PowerLab 4/35 model ML870; AD Instruments) and recorded with a data acquisition system (Chart 8 for Windows, version v8.1.11) using a standard PC desktop. The parameters evaluated were tidal volume, respiratory rate, and minute ventilation, and they were measured for a period of 60 s, with three repetitions.

Next, the airways were washed with 2 ml of ice-cold PBS for the collection of BALF and the lungs were removed, as previously described by Magalhães et al. (2015). The left lung was collected for morphometric analysis, and the right lung was removed, snap frozen in dry ice, and kept at −80°C until assayed.

### Inflammatory Infiltrate in the Lung

The BALF collected was processed, and the total and differential number of leukocytes was counted, as previously described by Magalhaes et al. (2018).

### Quantification of Eosinophil and Neutrophil Accumulation in the Lung

Quantification of eosinophil and neutrophil accumulation in the lung parenchyma was determined through measurements of pulmonary eosinophil peroxidase (EPO) (Magalhaes et al., 2018) and myeloperoxidase (MPO) activities (Barroso et al., 2017).

### Proteins Quantification by Western Blotting

ERK1/2 was measured by the Western blotting technique as described by Magalhaes et al. (2018). The measurement of total ERK1/2 and p-ERK1/2 was performed on the same membrane, following the protocol of stripping and reprobing the samples. Thus, we calculated the ratio between pERK1/2 and t-ERK1/2 in each sample. Staining was visualized and quantified on a LI-COR Odyssey Scanner (Lincoln, NE, United States).

### Morphometric Analysis of Mucus Deposition in the Airways

Lung was prepared for histological analysis, as previously described (Magalhães et al., 2015). Mucus deposition was evaluated by periodic acid–Schiff (PAS) staining in lung sections. For quantification, images were obtained from lung fields at ×20 (final magnification = ×200), as previously described by Reis et al. (2015). Results were expressed as the PAS-positive area (pixels/μm^2^).

### Morphometric Analysis of Extracellular Matrix Deposition in the Lung

For extracellular matrix deposition analysis, images were obtained from lung fields at ×20 (final magnification = ×200), as previously described (Magalhaes et al., 2018). Results are expressed as the percentage of extracellular matrix deposition/tissue area.

### Statistical Analysis

All results were expressed as mean ± SEM. Comparisons among groups were made by one-way ANOVA, followed by the post hoc Newman–Keuls test. All analyses and graphs were performed with the software GraphPad Prism (version 5.0, GraphPad Software, Inc., La Jolla, California, EUA). The level of significance was of *p* < 0.05.

## Results

### Effect of Ang-(1-7) Treatment on Mice Exploratory and Locomotor Activities

**FIGURE 2:**
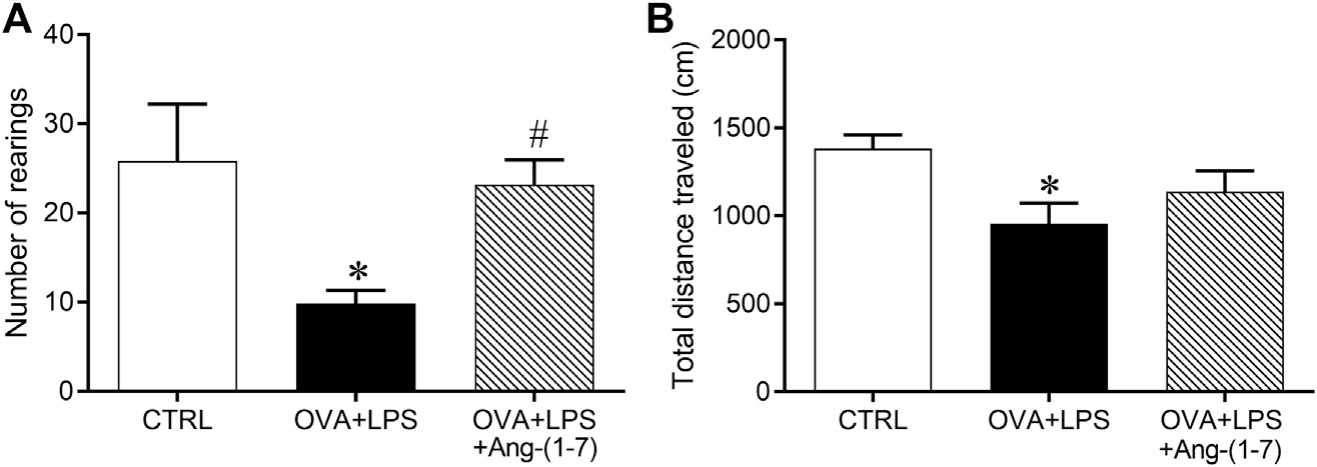
Number of rearings, evaluated by vertical elevation on hind legs **(A)** and total traveled distance in an open field arena (**B,** cm). Exploratory and locomotor activities were evaluated in control (*n* = 6), ovalbumin (OVA) and LPS challenge (OVA + LPS; *n* = 6), and OVA + LPS mice treated with Ang-(1-7)/HPBCD (60 µg/kg; *n* = 6). ^*^ *p* < 0.05 is relative to the CRTL group, and ^#^ *p* < 0.05 is relative to the OVA + LPS group (one-way ANOVA followed by Newman–Keuls).

As expected, challenge with OVA + LPS induced a decrease in both the exploratory (10 ± 1 rearings vs. 26 ± 6 rearings in the CTRL group; Figure 2A) and locomotor (955 ± 118 cm vs. 1,382 ± 79 cm in the CTRL group; Figure 2B) activities. Treatment with Ang-(1-7) attenuated the alteration in these behaviors induced in animals subjected to OVA + LPS (Figure 2).

### Effect of Ang-(1-7) Treatment on Lung Function

**FIGURE 3:**
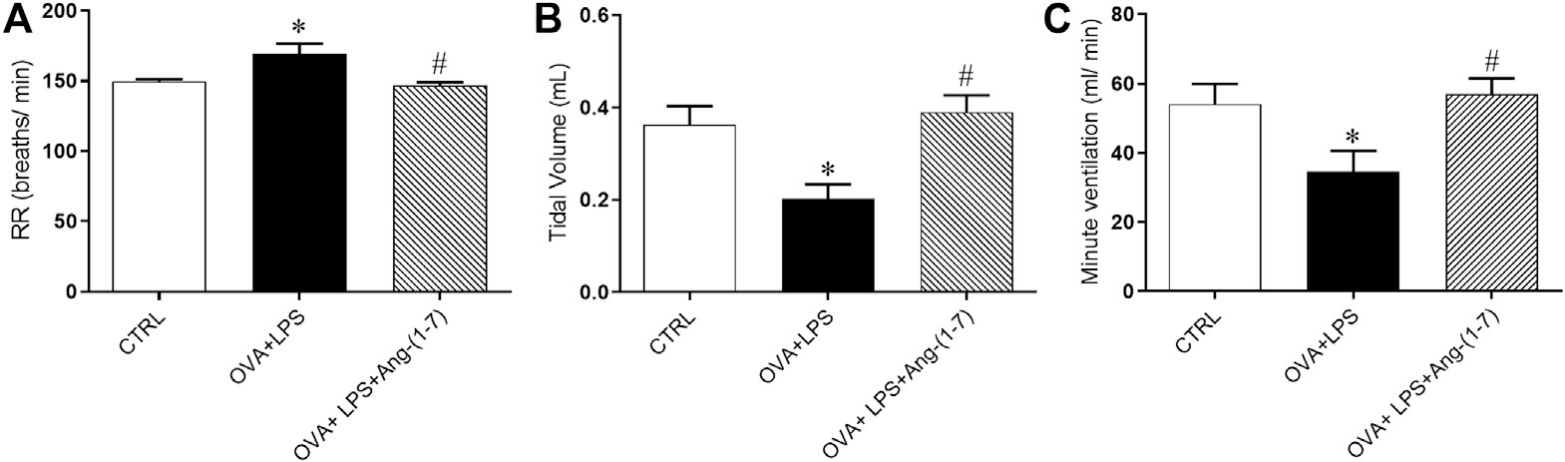
Effect of Ang-(1-7) treatment on pulmonary function. **(A)** Respiratory rate (RR, breaths/min), **(B)** tidal volume (V_T_, ml), and **(C)** minute ventilation (V_E_, ml/min) in control (*n* = 5), ovalbumin (OVA) and LPS challenge (OVA + LPS; *n* = 5), and OVA + LPS mice treated with Ang-(1-7)/HPBCD (60 µg/kg; *n* = 5). ^*^ *p* < 0.05 is relative to the CRTL group, and ^#^ *p* < 0.05 is relative to the OVA-LPS group (one-way ANOVA followed by Newman–Keuls).

OVA + LPS-challenged mice presented significant elevated respiratory rate (RR, breaths/min, Figure 3A) and decreased tidal volume (VT, ml, Figure 3B) in relation to the CTRL group. Ang-(1-7) treatment normalized these changes (Figures 3A,Figures 3B). The minute ventilation (VE, ml/min, Figure 3C) was decreased in OVA + LPS-challenged mice in relation to CTRL and OVA + LPS + Ang-(1-7) groups.

### Treatment With Ang-(1-7) Decreased Eosinophil and Neutrophil Accumulation in the Lung

**FIGURE 4:**
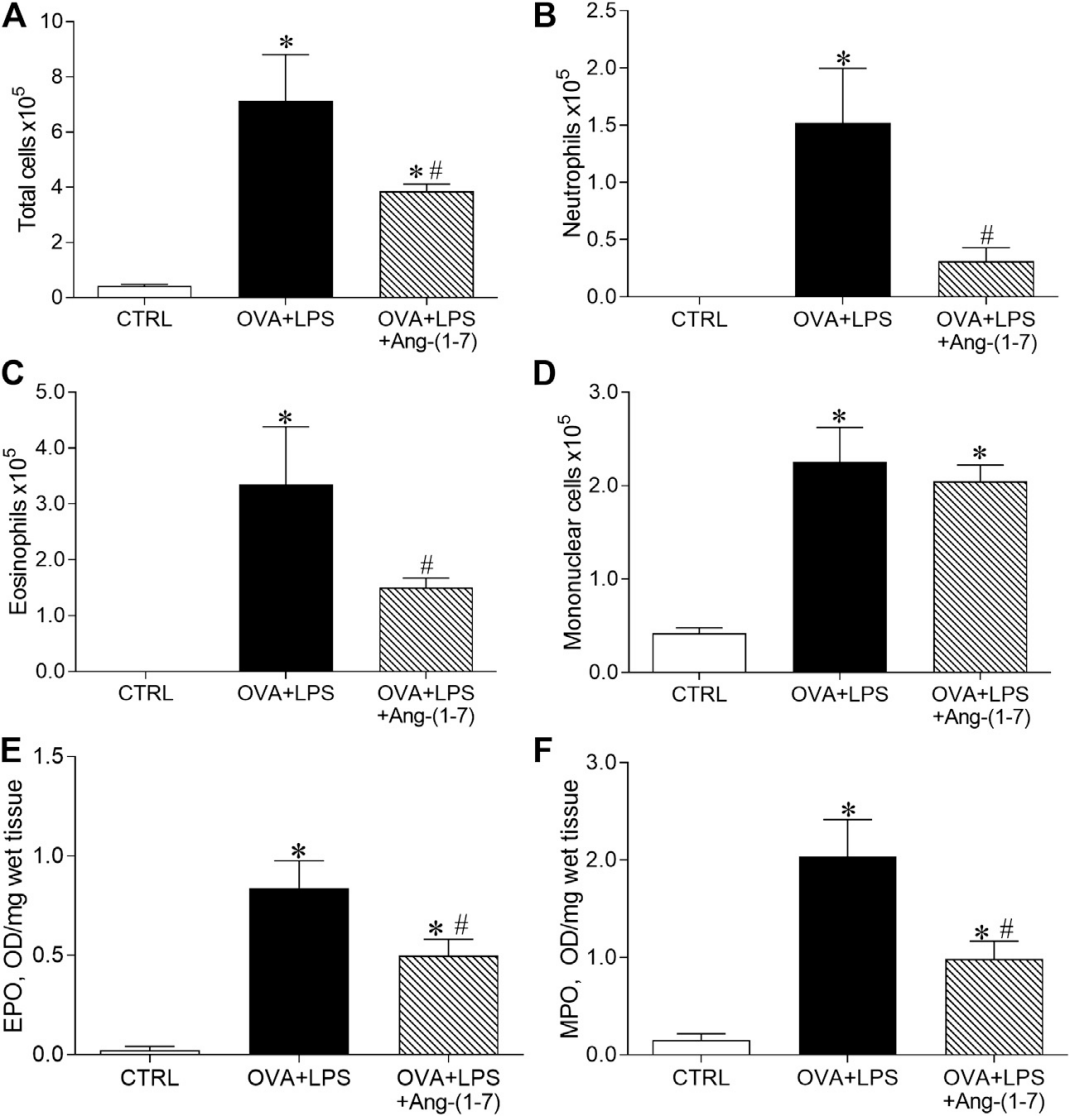
Quantification of cells in the bronchoalveolar lavage fluid. **(A)** Total cells; **(B)** neutrophils; **(C)** eosinophils; **(D)** mononuclear cells. Measurement of eosinophil peroxidase (EPO; **E**) and myeloperoxidase (MPO; **F**) enzymatic activities in control (CTRL, *n* = 5), ovalbumin (OVA) and LPS challenge (OVA + LPS; *n* = 5), and OVA + LPS mice treated with Ang-(1-7)/HPBCD (60 µg/kg; *n* = 6). ^*^ *p* < 0.05 is relative to the CRTL group, and ^#^ *p* < 0.05 is relative to the OVA-LPS group (one-way ANOVA followed by Newman–Keuls).

The OVA + LPS group presented increased number of total cells (Figure 4A), neutrophils (Figure 3B), eosinophils (Figure 4C), and mononuclear cells (Figure 3D) compared to the CTRL group. Mice from the OVA + LPS + Ang-(1-7) group showed attenuated number of total cells, neutrophils, and eosinophil, as compared to the OVA + LPS group (Figure 4). However, there was no significant difference in mononuclear cells in OVA + LPS mice treated with Ang-(1-7) (Figure 4D). Corroborating these results, similar changes were observed in EPO (Figure 3E) and MPO (Figure 4F) activities in the lung.

### Treatment With Ang-(1-7) Decreased Mucus and Extracellular Matrix Deposition in the Airway

**FIGURE 5:**
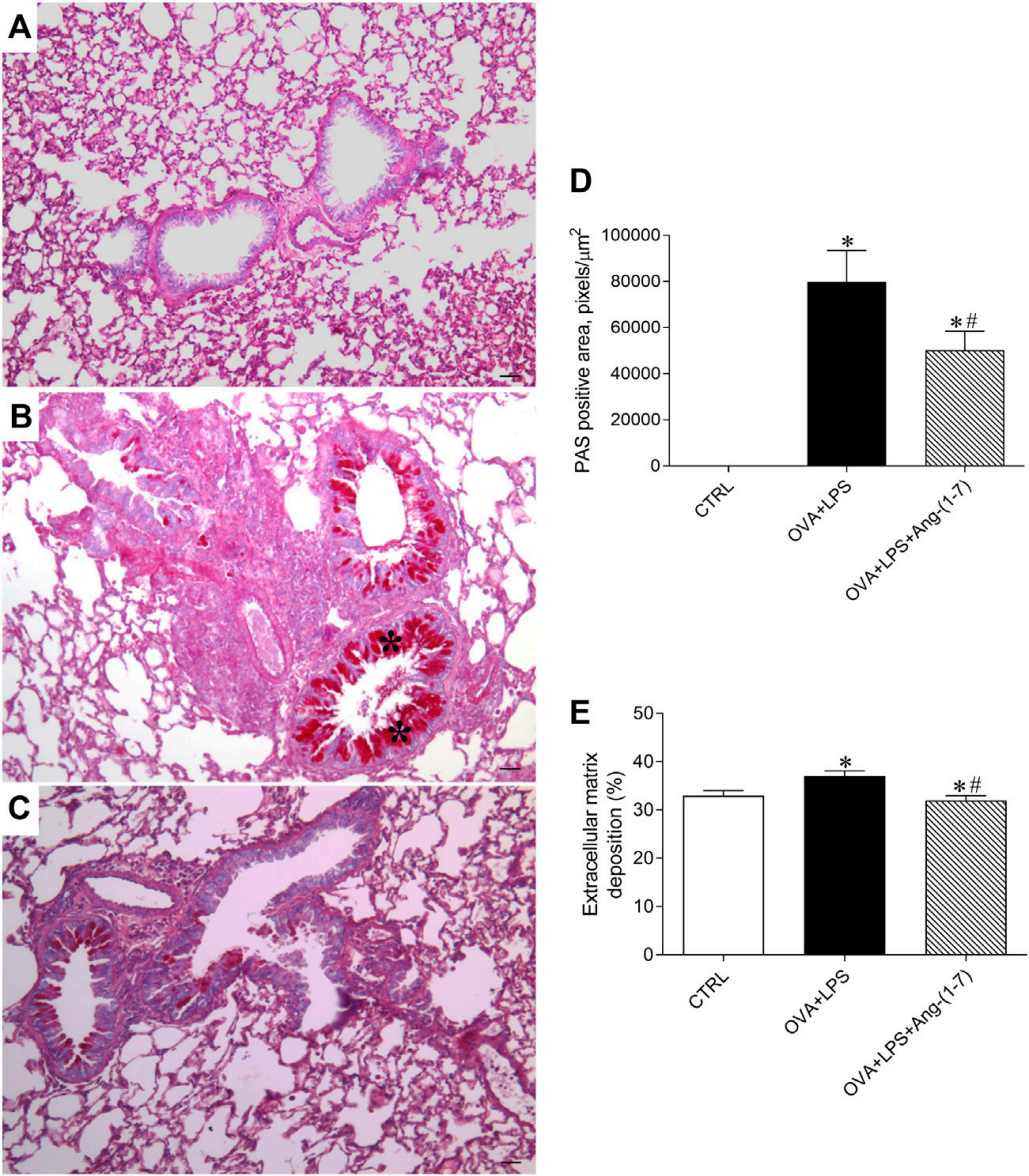
Evaluation of mucus deposition in the airways. Representative images of lung sections stained with PAS in control (CTRL; **A**), ovalbumin (OVA) and LPS challenge (OVA + LPS; **B**), and OVA + LPS mice treated with Ang-(1-7)/HPBCD (60 µg/kg; **C**). As can be seen, OVA + LPS-challenged mice presented marked mucus deposition in the airway (asterisks in **B**) compared to CTRL mice **(A)**, which was greatly attenuated by Ang-(1-7) treatment. **(D)** Quantification of the periodic acid–Schiff (PAS) stain positive area in the lungs (*n* = 5 each). **(E)** Extracellular matrix deposition in the lung (%; *n* = 5–6). ^*^ *p* < 0.05 is relative to the CRTL group, and ^#^ *p* < 0.05 is relative to the OVA-LPS group (one-way ANOVA followed by Newman–Keuls).

Mucus deposition was evaluated by PAS staining in lung sections, as shown in Figures 5A–C. Morphometric analysis of the area marked for PAS staining showed that mice of the OVA + LPS group presented an increased amount of mucus in the airways (Figure 5D). In contrast, OVA + LPS mice treated with Ang-(1-7) showed a lower area of PAS staining in comparison to nontreated animals (Figure 5D). Additionally, animals treated with Ang-(1-7) presented a similar percentage of extracellular matrix deposition in the lung as control mice and significantly lower than OVA + LPS mice (Figure 5E).

### Ang-(1-7) Treatment Reduced ERK1/2 Phosphorylation in the Lung

**FIGURE 6:**
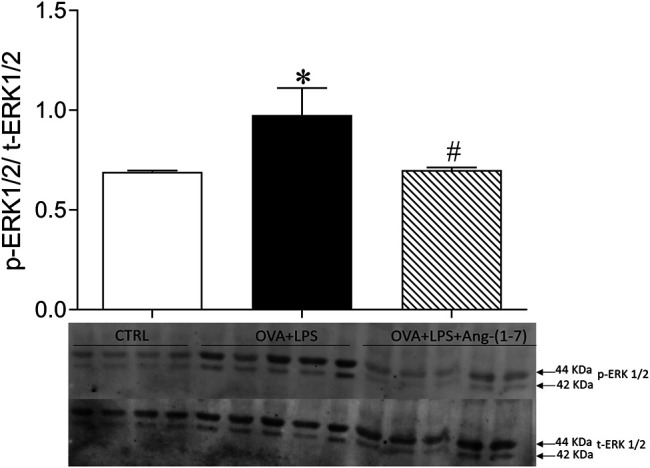
Ratio of phosphorylated and total ERK1/2 quantified by Western blotting in the lung of CTRL (*n* = 4), OVA + LPS (*n* = 5), and OVA + LPS + Ang-(1-7) (*n* = 5). The graph is representative blots illustrating molecular weight of each band in KDa. ^*^ *p* < 0.05 in comparison to CTRL, and ^#^ *p* < 0.05 in comparison to Ang-(1-7) treatment (one-way ANOVA followed by the Newman–Keuls test).

As shown in Figure 6A, OVA + LPS induced an increase in phosphorylation of ERK1/2, whereas treatment with Ang-(1-7) blunted this effect. This result suggests a mechanism for Ang-(1-7) in the resolution of eosinophilic and neutrophilic inflammation, i.e., suppressing ERK1/2 in the lung.

## Discussion

In this study, treatment with Ang-(1-7) at the peak of inflammation of eosinophils and neutrophils resulted in the following: 1) increased the exploratory and locomotor activities; 2) normalized respiratory rate, tidal volume, and minute ventilation; 3) reduced the accumulation of eosinophils and neutrophils in the lung, without a significant change in the number of mononuclear cells; 4) decreased the activity of EPO and MPO in the lung; 5) decreased the deposition of mucus and extracellular matrix in the airways; and 6) reduced the phosphorylation of ERK1/2 in the lung. These data demonstrate a therapeutic effect of Ang-(1-7) in a model of eosinophilic and neutrophilic asthma.

Eosinophilic and neutrophilic airway inflammation has been linked to the severe, chronic, and acute forms of asthma, such as steroid-insensitive asthma and acute exacerbation (Green et al., 2002; Felton et al., 2014; Jasper et al., 2019; Hadjigol et al., 2020). Since eosinophils and neutrophils are involved in asthma exacerbations, the resolution of lung inflammation depends on reducing the number and activity of these cells in the lungs. Here, we showed that Ang-(1-7) attenuated eosinophilic and neutrophilic inflammation in an experimental model of asthma induced by OVA and LPS. These data are in agreement with previous studies of our group that showed in an OVA-induced asthma, an eosinophilic model, that treatment with Ang-(1-7) reduced the accumulation of eosinophils in the lungs, increased the number of apoptotic eosinophils, and induced the lung to return to homeostasis (Magalhaes et al., 2018). In the present study, Ang-(1-7) administration at the peak of both eosinophilic and neutrophilic inflammation led to a significant reduction in the number of eosinophils and neutrophils in the lung, associated with reduced activity of EPO and MPO enzymes.

The fact that the number of mononuclear cells was not affected is an additional indication that Ang-(1-7) has a proresolving effect. Macrophages are important for the clearance of apoptotic cells, and this phenomenon is essential for the resolutive process (Perez et al., 2014). In addition, we have previously shown that Ang-(1-7) increased the efferocytosis capacity of macrophages (Barroso et al., 2017; Magalhaes et al., 2018). The results of the present study strengthen these previous results and show the ability of Ang-(1-7) to activate the resolution of pulmonary inflammation by reducing the number and activity of two different effector cells, in exacerbated asthma. Lowe et al. (2015) showed that treatment with corticoid in guinea pigs with allergic pulmonary inflammation induced by OVA reduced eosinophils and neutrophils in the lungs. However, these effects were not seen in mice challenged with OVA + LPS, which suggests a corticoid inability to modulate inflammation in exacerbated asthma. Similar results were also observed by Hadjigol et al. (2020).

It has been demonstrated that ERK1/2 activation regulates GATA3 stability, Th2 differentiation, and mast cell and eosinophil activities (Alam and Gorska, 2011). In addition, EK1/2 is activated in airway epithelial cells, macrophages, and neutrophils exposed to LPS (Boots et al., 2012; Shi et al., 2017; Zhou et al., 2018). In a sense, many asthma-related cytokines and chemokines have been shown to signal through an ERK1/2-dependent pathway (Alam and Gorska, 2011). Different studies point to an inhibitory effect of Ang-(1-7) on ERK1/2 phosphorylation, and it is believed that this could be one of the mechanisms underlying the anti-inflammatory and proresolving effects of this peptide (Magalhães et al., 2015; Magalhaes et al., 2018; Santos et al., 2018). El-Hashim et al. (2012) showed that OVA challenge significantly increased lung levels of p-ERK1/2 (El-Hashim et al., 2012). In addition, these authors showed that treatment with Ang-(1-7) significantly inhibited the increase in ERK1/2 in OVA challenge mice (El-Hashim et al., 2012). Furthermore, the Ang-(1-7) effect was reversed by cotreatment with A779, a Mas receptor antagonist (El-Hashim et al., 2012). In a previous study, we have shown that continuous treatment with Ang-(1-7) during the period of OVA challenge was associated with a reduction in p-ERK1/2 in the lungs of asthmatic animals (Magalhães et al., 2015). Additionally, we found that Mas receptor knockout mice challenged with OVA had an increase in p-ERK1/2 in the lungs, when compared to WT-OVA (Magalhaes et al., 2018). Finally, in a short-term model of asthma, we reported a reduction in ERK1/2 activation in the lung when Ang-(1-7) was given 24 h after challenge (Magalhaes et al., 2018). It has been argued that ACE2/Ang-(1-7)/Mas activation has protective effects on LPS-induced lung injury (Li et al., 2015; Li et al., 2016; Cao et al., 2019). These effects can be associated, at least in part, with suppression of ERK1/2 phosphorylation (Li et al., 2015; Li et al., 2016). Sawatzky et al. (2006) showed that specific inhibition of ERK1/2 induces leukocyte apoptosis and accelerates inflammation resolution (Sawatzky et al., 2006). The results of the present study showed a significant lower p-ERK1/2/t-ERK1-2 ratio in the OVA-LPS group treated with Ang-(1-7). This finding reinforces the hypothesis that attenuation of ERK1/2 phosphorylation is one proresolutive mechanism regulated by the Ang-(1-7)/Mas pathway in asthma.

In asthma, especially when exacerbated, there is an increase in the production of mucus in the airways (Evans et al., 2009; Dunican et al., 2018; Martínez-Rivera et al., 2018). In addition, mucus hypersecretion is a significant marker of disease severity (Dunican et al., 2018; Martínez-Rivera et al., 2018). An excess of mucus obstructs the airways and contributes to bronchial hyperresponsiveness (Martínez-Rivera et al., 2018). In the small human airways and in all mice intrapulmonary airways, there are few or no visible “mucus” or “chalice” cells by histological staining at baseline conditions (Evans et al., 2009), as observed in our study in the control group. However, in the OVA + LPS group, mucus production exceeded depuration rate leading to a visible intracellular accumulation in the PAS stain. In addition, the accumulation of mucus was accompanied by an increase in the respiratory rate, reduced tidal volume, and minute ventilation. Administration of Ang-(1-7) at the peak of eosinophilic and neutrophilic inflammation led to a significant decrease in mucus deposition and improvement in lung function in OVA-LPS mice. Similar results were observed for extracellular matrix deposition.

In line with these findings, Ang-(1-7) also improved the functional capacity of OVA + LPS mice. Asthmatic individuals tend to have lower exercise tolerance due to a certain degree of airway obstruction at rest, exercise-induced bronchospasm, decreased ventilatory capacity, increased sensation of dyspnea, anxiety, and depression (Boudreau et al., 2014; Jayasinghe et al., 2015; Cordova-Rivera et al., 2018). Among the tests to assess mice activity, the open field test is designed to evaluate locomotion, anxiety-like, and exploratory behaviors (Tatem et al., 2014). The OVA + LPS group showed a significant reduction in the rearing activity and total distance traveled, which was not seen in OVA + LPS mice treated with Ang-(1-7). A role for Ang-(1-7) in exercise performance was suggested by previous studies. Genetic deletion of the Mas receptor, both in models of cardiac (Guimarães et al., 2012) and lung injury (Magalhães et al., 2016), leads to worse results in the incremental exercise test. In addition, we have previously shown that Ang-(1-7) improved horizontal and vertical locomotor activities in a model of pulmonary emphysema (Bastos et al., 2020).

The findings of the present study showed that oral administration of Ang-(1-7) can be valuable for the treatment of neutrophil- and eosinophil-mediated asthma. Current therapies are unable to adequately control airway inflammation, increasing the chances of exacerbations and death, since asthma exacerbations are often severe and difficult to control. It is important to emphasize that poor asthma control associated to an increased rate of exacerbations establishes a constant and intense inflammatory process that, consequently, induces tissue remodeling and the decline in lung function. Activation of the ACE2/Ang-(1-7)/Mas axis proved to be a promising approach to promote the resolution of pulmonary inflammation and to contribute to the return of tissue homeostasis. Therefore, clinical studies are needed to assess the therapeutic potential of Ang-(1-7) in patients with different asthma phenotypes.

## Data Availability

The data that support the findings of this study are available from the corresponding authors upon reasonable request.
